# Comprehensive analyses of *ZFP* gene family and characterization of expression profiles during plant hormone response in cotton

**DOI:** 10.1186/s12870-019-1932-6

**Published:** 2019-07-23

**Authors:** Peng He, Yan Yang, Zihua Wang, Peng Zhao, Yi Yuan, Li Zhang, Yueqin Ma, Chaoyou Pang, Jianing Yu, Guanghui Xiao

**Affiliations:** 1 College of Life Sciences, Shaanxi Normal University, Xi’an, 710119 China; 2 Institute for Advanced Studies, Wuhan University, Wuhan, 430072 China; 3 State Key Laboratory of Cotton Biology, Institute of Cotton Research of Chinese Academy of Agricultural Sciences, Anyang, 455000 China; 4 Zhengzhou Research Base, State Key Laboratory of Cotton Biology, Zhengzhou University, Zhengzhou, 450001 China; 5 Key Laboratory of the Ministry of Education for Medicinal Plant Resources and Natural Pharmaceutical Chemistry, National Engineering Laboratory for Resource Development of Endangered Crude Drugs in the Northwest of China, College of Life Sciences, Shaanxi Normal University, Xi’an, 710119 China

**Keywords:** Cotton, Zinc finger proteins, Plant hormone, Expression patterns, Fiber development

## Abstract

**Background:**

Zinc finger proteins (ZFPs) containing only a single zinc finger domain play important roles in the regulation of plant growth and development, as well as in biotic and abiotic stress responses. To date, the evolutionary history and functions of the *ZFP* gene family have not been identified in cotton.

**Results:**

In this paper, we identified 29 *ZFP* genes in *Gossypium hirsutum*. This gene family was divided into seven subfamilies, 22 of which were distributed over 17 chromosomes. Bioinformatic analysis revealed that 20 *GhZFP* genes originated from whole genome duplications and two originated from dispersed duplication events, indicating that whole genome duplication is the main force in the expansion of the *GhZFP* gene family. Most *GhZFP8* subfamily genes, except for *GhZFP8–3,* were highly expressed during fiber cell growth, and were induced by brassinosteroids in vitro. Furthermore, we found that a large number of *GhZFP* genes contained gibberellic acid responsive elements, auxin responsive elements, and E-box elements in their promoter regions. Exogenous application of these hormones significantly stimulated the expression of these genes.

**Conclusions:**

Our findings reveal that *GhZFP8* genes are involved in cotton fiber development and widely induced by auxin, gibberellin and BR, which provides a foundation for the identification of more downstream genes with potential roles in phytohormone stimuli, and a basis for breeding better cotton varieties in the future.

**Electronic supplementary material:**

The online version of this article (10.1186/s12870-019-1932-6) contains supplementary material, which is available to authorized users.

## Background

Cotton is one of the most important crops globally for industrial fiber and oil seed production. Cotton fiber, a primary resource for the textile industry, contributes more than thirteen billion dollars annually to the global economy and creates 330 million jobs in the agricultural or industrial sectors [1]. *Gossypium hirsutum* (*G. hirsutum*), the most commonly cultivated cotton species for fiber and oil, accounts for more than 90% of annual global cotton production [2]. Cotton is composed of both diploid and tetraploid species belonging to the *Gossypium* genus. *G. hirsutum* is an AADD allotetraploid species, which evolved from A-genome diploids resembling *Gossypium arboreum* and D-genome diploids resembling *Gossypium raimondii,* approximately 1–2 million years ago (MYA) [2].

Previous research has shown that phytohormones play critical roles in the process of fiber development. For example, it has been reported that endogenous levels of gibberenllic acid (GA_3_) are higher in a long staple cotton cultivar compared to medium and short staple cultivars [3]. Exogenous application of GA_3_ improved fiber elongation, increased fiber cell wall thickness and increased the weight of individual fibers [3, 4]. In vitro ovule culture assay showed that brassinosteroid (BR) significantly promoted fiber elongation, whereas its biosynthesis inhibitor brassinazole (BRZ) abrogated fiber elongation [5]. Treatment with BR and BRZ increased and decreased the expression of cell wall related genes, respectively. In addition, genes involved in the BR biosynthetic pathway were up-regulated during fiber initiation and elongation stages [6, 7]. Exogenous application of indole-3-acetic acid (IAA) significantly increased the total fiber volume [8], while application of IAA transport inhibitor, 1-naphthylphthalamic acid (NPA), dramatically reduced IAA content and the number of fiber cells [9]. Overexpression of the IAA biosynthetic gene *iaaM* significantly increased fiber cell number, final yield, and overall quality [9].

Zinc-finger proteins (ZFPs) have been shown to act a pivotal part in diverse biological processes, and can be divided into 23 subfamilies based on their structural differences, including A20, AN, Bbox, CDGSH, CHY, DHHC, Dof, FYVE, GATA, LYAR, MSRING, NFX1, PADPP, PHD, RBPO, Ring, TAZ, TDDP, TFIIB, Ubox, UBR, WRKY, and ZK [10–12]. Although ZFPs are abundant in plants, only a few ZFPs, which contain only single zinc finger domain, have been characterized to function in regulation of plant height [13, 14], plant development [15], secondary cell wall thickening [16], anther development [17], root development [18], flower development [19], seed germination [20], and fruit ripening [21].

*ZFP* genes have been found to participate in various biological processes, including signal transduction, transcriptional regulation, RNA binding and morphogenesis, and stress response [22–24]. In *Arabidopsis*, ZFP proteins have been devided into 9 groups: C2H2, C8, C6, C3HC4, C2HC, C2HC5, C4, C3H and C4HC3 [25]. *AtZFP1*, was highly expressed in apical meristem, vascular system, and seedlings at three days post germination. The *zfp1* mutant has a dominant phenotype in leaf initiation [26]. *ZFP6* encoded a C2H2 zinc finger protein, which are involved in regulating trichome development by integrating gibberellic acid and cytokinin signaling [27]. *ZFP5*, a gene downstream of *ZFP6* signaling, encodes a cell-to-cell mobile mRNA, necessary for regulating trichome development [27]. *ZFP10*, a DNA binding transcription factor that targets specific sequences, was found to be involved in zinc ion and nucleic acid binding [28]. Overexpression of *ZFP11* resulted in mortality and a deformed phenotype in *Arabidopsis* [29].

In this study, we identified 29 *GhZFP* genes in *G. hirsutum* and charactered their evolutionary relationships, chromosomal distribution, gene duplication and gene structure to gain insight into the role of *GhZFPs* in cotton. Gene expression patterns showed that *GhZFP8* subfamily genes, except for *GhZFP8–3,* were significantly expressed during fiber cell development. Exogenous application of BR enhanced the transcription level of these genes. In addition, we found that a large number of *GhZFP* genes contained gibberellic acid responsive element (GARE), auxin responsive element (AuxRE), or E-box element in their promoter regions. Our results showed that the majority of *GhZFP* genes containing these elements in their promoters were significantly up-regulated by exogenous application of gibberellic acid, auxin, and BR, respectively.

## Methods

### Plant materials and growth conditions

*Gossypium hirsutum* (Xuzhou 142) was grown in a climate-controlled greenhouse with a 16 h light and 8 h dark cycle at 30 °C, as previously reported [30]. For phytohormone treatment experiments, a total of 30 ovules were used for each treatment and three biological triplicates were performed for each experiment.

### Sequence retrieval, multiple sequence alignment, and phylogenetic analysis

The cotton genome sequences were acquired from the CottonGen website (https://www.cottongen.org). The *Arabidopsis* genome sequences were downloaded from TAIR 10 (http://www.arabidopsis.org). HMMER software with default parameters was used to search for corresponding protein sequences using the conserved ZFP domain as a query. We used the BLAST program to further identify ZFP sequences based on homology. Multiple sequence alignments of all identified ZFPs were performed using Clustal X [31]. A phylogenetic tree of deduced amino acid sequences was constructed using the neighbor-joining algorithm with default parameters and 1000 bootstrap replicates in MEGA 6.0 (https://www.megasoftware.net).

### Analysis of chromosomal location, gene structure and conserved motif

The positional information for *GhZFP* was obtained from the parsed general feature format (GFF) files downloaded from the CottonGen website. For the exon-intron structural analysis of *GhZFP* genes, the coding sequences were used to align with their genomic DNA sequences and the structure diagrams were drawn using the online Gene Structure Display Server (GSDS) program (http://gsds.cbi.pku.edu.cn/). Conserved motifs of GhZFP proteins were investigated using the online toolkit Multiple Expectation maximization for Motif Elicitation (MEME 3.0.3; http://meme-suite.org/). The optimized parameters of MEME were employed as follows: number of repetitions, any; maximum number of motifs, 50; and the optimum width of each motif, between 6 and 300 residues, and retaining only motifs associated with an *E* value <e^− 5^. The identified protein motifs were further annotated with ScanProsite (http://prosite.expasy.org/scanprosite/).

### RNA extraction and quantitative RT-PCR (qRT-PCR) analysis

Cotton ovules harvested after phytohormone treatment for each indicated time were frozen in liquid nitrogen and then ground into fine powder with a mortar and pestle using a previously described method [1]. The total RNA was extracted using a PureLink™ RNA mini kit (Invitrogen, Lot no.1687455) according to the manufacturer’s instructions, and cDNA was reverse-transcribed from 1.2 μg total RNA [32]. In the qRT-PCR experiments, each gene was run in three biological replicates and three technical replicates with the reaction parameters as follows: 95 °C for 10 min, followed by 40 cycles of 95 °C for 10 s and 56 °C for 30 s. A melting curve was generated from 65 to 95 °C. Cotton *GhUBQ7* (GenBank no. AY189972) was used as the internal control. Primers for qRT-PCR analysis are listed in Additional file 1: Table S3. SigmaStat software was used for one-way statistical variance analysis.

### In vitro ovule culture

The in vitro ovule culture was performed according to a previously published method [26]. Cotton ovules collected at 1 day post anthesis (DPA) were sterilized in 10% sodium hypochlorite and cultured in medium at 30 °C [33]. Five Micrometre 1-Naphthylacetic acid (NAA, Sigma), 1 μM gibberellin acid (GA_3_, Sigma) and 5 μM BR (Sigma) were added to the culture medium for the indicated time, respectively. After treatment, samples were collected for qRT-PCR experiments.

### Identification of *cis*-elements in *GhZFP* promoter region

The predicted promoter sequences of *GhZFP* were downloaded from the CottonGen website (https://www.cottongen.org). The *cis*-elements in *GhZFP* promoter regions were predicted using the website Plant Cis-acting Regulatory DNA Elements (PLACE, https://www.dna.affrc.go.jp/PLACE/?action=newplace) [34].

## Results

### Genome-wide identification of the *ZFP* gene family in *Gossypium*

Whole genome sequences of three sequenced cotton species (*G. hirsutum*, *G. arboreum* and *G. raimondii*) were used to identify the ZFP proteins. *Arabidopsis* ZFP protein sequences were used as queries to search the three reference genomes to screen out candidate ZFP proteins in cotton. Using HMMR software for further selection of ZFP candidates based on conserved domains, we identified 29 ZFPs in *G. hirsutum*, along with 23 in *G. arboreum* and 23 in *G. raimondii*. Among the 29 GhZFP proteins found in *G. hirsutum* genome, 13 members originated from the At sub-genome and 16 from the Dt sub-genome (Additional file 1: Table S1). The lengths of GhZFP proteins ranged from 170 (GhZFP10–9) to 295 (GhZFP10–1) amino acids (aa), with an average length of 238 aa. The GaZFP proteins ranged from 165 (GaZFP11–3) to 295 (GaZFP10–1) amino acids, with an average length of 226 aa. The GrZFP proteins ranged from 116 (GrZFP4–1) to 490 (GrZFP10–1) amino acids with an average length of 272 aa. The physicochemical parameters analysis showed that the molecular weight of GhZFP proteins ranged from 21.49 (GhZFP8–1) to 33.23 (GhZFP10–2) KDa with an average value of 26.42 KDa and the isoelectric point (pl) of GhZFP proteins ranged from 5.12 (GhZFP4–1) to 9.34 (GhZFP11–1) with an average value of 6.90 (Table 1).

**Table 1:** Physicochemical parameters of 29 *GhZFP* genes in *G. hirsutum* genome

Name	Protein length	Protein MW (kD)	Protein pI	Extinction coefficient	Instability index	Aliphatic index
GhZFP1–1	250	27.65	5.45	0.49	41.23	77.36
GhZFP2–1	253	28.22	7.79	0.77	57.66	61.7
GhZFP2–2	293	32.00	8.29	1.02	44.54	59.32
GhZFP3–1	253	28.15	8.28	0.77	56.71	63.24
GhZFP3–2	292	31.94	8.28	1.07	45.78	58.87
GhZFP4–2	250	27.06	5.45	0.49	41.23	77.36
GhZFP5–1	239	26.56	6.11	0.67	61.39	49.04
GhZFP5–2	239	26.56	6.11	0.67	61.39	49.04
GhZFP6–1	205	21.60	8.93	0.43	53.39	61.56
GhZFP7–1	250	27.05	5.96	0.43	44.25	59.4
GhZFP8–1	194	21.49	9.07	1.07	69.61	44.79
GhZFP10–10	222	24.97	6.83	1.13	56.11	53.24
GhZFP10–12	270	29.22	6.29	1.07	58.02	65.78
GhZFP4–1	250	27.06	5.12	0.43	39.92	78.92
GhZFP8–2	248	27.53	6.92	0.78	59.24	49.96
GhZFP8–3	235	26.28	6.19	0.77	59.79	46.13
GhZFP8–4	194	21.49	9.07	1.07	69.61	44.79
GhZFP10–1	295	33.10	6.17	1.37	56.79	56.2
GhZFP10–2	295	33.23	6.12	1.46	57.52	58.85
GhZFP10–3	206	23.33	6.99	1.39	64.01	65.39
GhZFP10–4	243	27.91	7.1	1.32	60.71	46.17
GhZFP10–5	206	23.33	6.99	1.39	64.01	65.39
GhZFP10–6	215	23.65	5.73	1.26	61.54	64.98
GhZFP10–7	215	23.65	5.73	1.26	61.54	64.98
GhZFP10–8	206	23.33	6.99	1.39	64.01	65.39
GhZFP10–9	170	23.33	6.99	1.39	64.01	65.39
GhZFP10–11	274	29.61	6.03	1.05	57.64	69.12
GhZFP10–13	215	23.65	5.73	1.26	61.54	64.98
GhZFP11–1	207	23.19	9.34	1.04	63.08	56.52

### Phylogenetic analysis, chromosomal location and gene duplication of the *ZFP* gene family

**Fig. 1:**
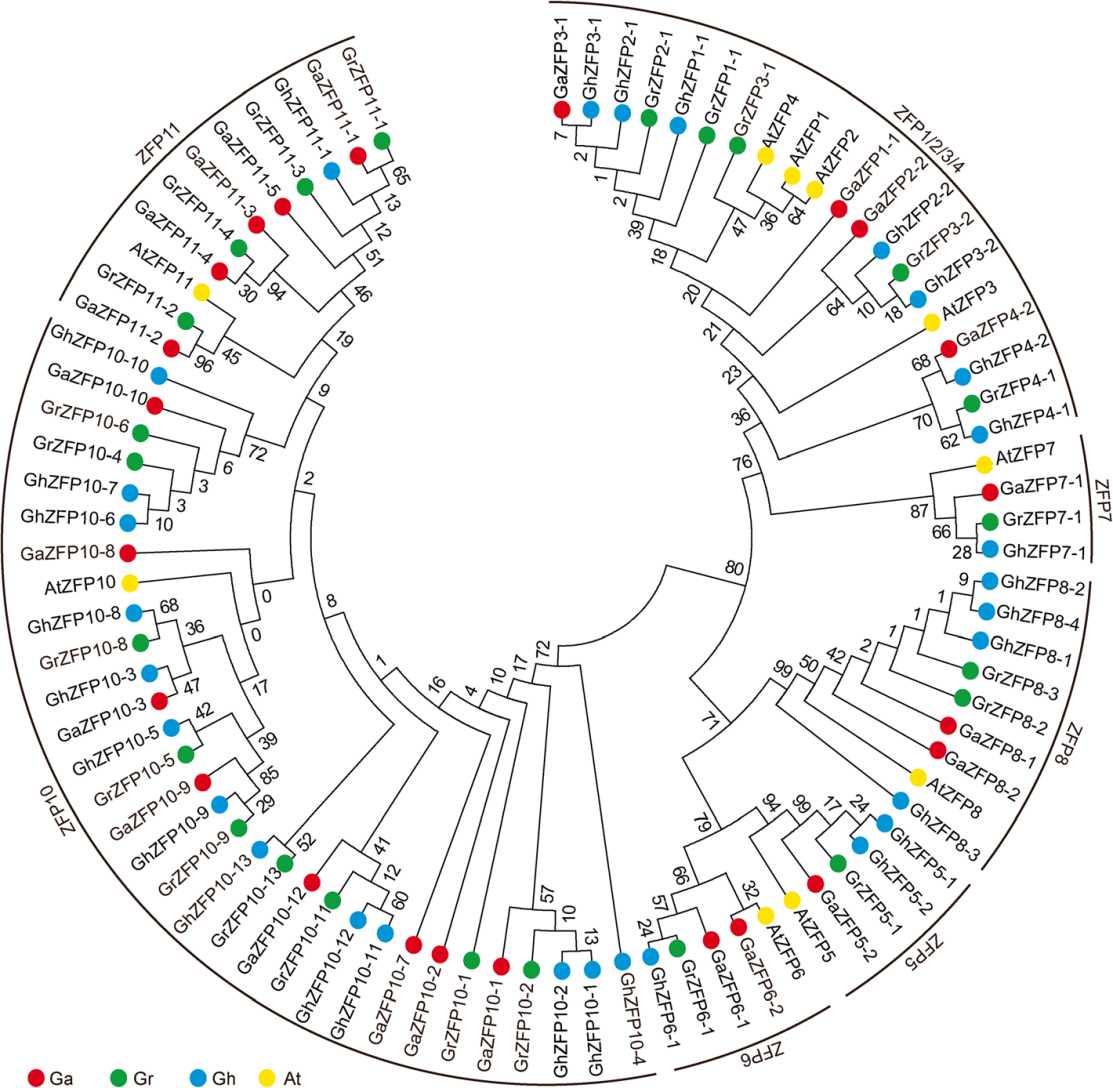
Phylogenetic analysis of the *ZFP* gene family**.** The phylogenetic tree was constructed using the full length ZFP protein amino acid sequences from *G. arboreum*, *G. raimondii*, *G. hirsutum* and *A. thaliana*. MEGA 6.0 software was used with the neighbor-joining method and bootstrapping with 1,000 iterations. At, *Arabidopsis thaliana*; Ga, *Gossypium arboreum*; Gr, *Gossypium raimondii*; Gh, *Gossypium hirsutum*

To gain further insights into the evolutionary history and phylogenetic relationships of the *ZFP* gene family, a neighbor-joining phylogenetic tree was constructed. Our results showed that *ZFP* family genes were clustered into seven subfamilies and that most of the orthologous genes between the diploids and the corresponding allotetraploid were grouped into the same clade (Fig. 1). As shown in phylogenetic tree (Fig. 1), a similar organization for cotton and *Arabidopsis* ZFP proteins and some orthologous relationships between both species were identified. Based on this analysis, cotton ZFP proteins were named based on their relationships to known *Arabidopsis* ZFPs. To validate this result from the neighbor-joining (NJ) method, we reconstructed the phylogenetic tree of *ZFP* genes using the maximum likelihood method and again found that *ZFP* genes were divided into seven subfamilies (Additional file 2: Figure S1), similar to the result obtained using the neighbor-joining (NJ) method.

In contrast to *Arabidopsis*, two *GaZFP8* and eight *GaZFP10* genes were found in *G. arboreum*, as well as two *GrZFP8* and nine *GrZFP10* genes in *G. raimondii*. Furthermore, four *TcZFP8* genes and two *TcZFP10* genes were found in *T. cacao* (Additional file 3: Figure S2). To validate the evolutionary relationship of ZFPs, we introduced the ZFP proteins from *O. sativa* and *G. max*. As shown in Additional file 4: Figure S3, ZFP proteins were also clustered into seven subfamilies, consistent with our previous results (Additional file 4: Figure S3). Comparison of gene number of *ZFP8* and *ZFP10* genes in three *Gossypium* species and *Arabidopsis* suggests that the expansion of these gene subfamilies may have occurred in *Gossypium* after divergence from the common ancestor of *Arabidopsis* and *Gossypium*.

**Fig. 2:**
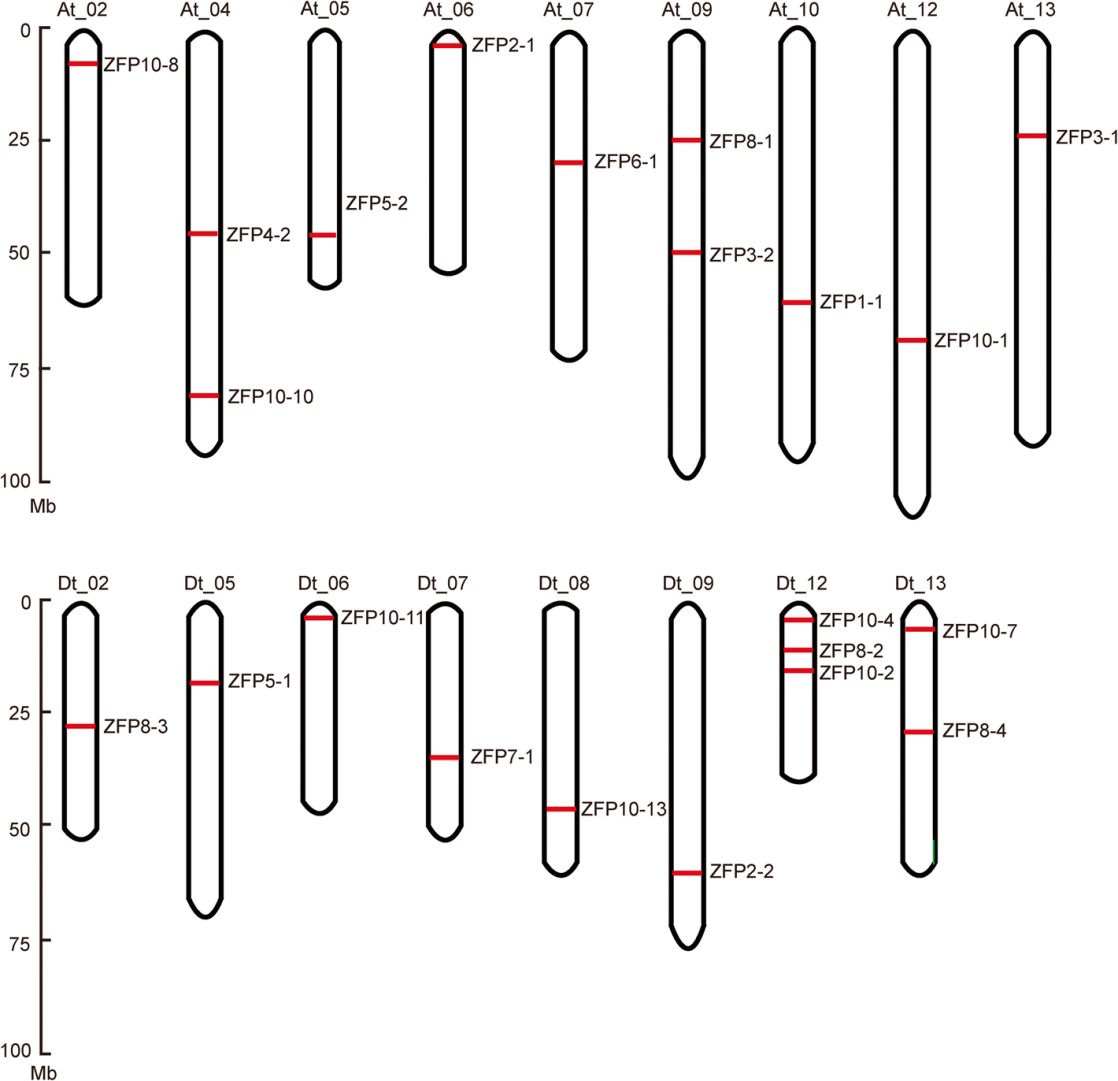
Chromosomal location of *GhZFP* genes on 17 chromosomes**.** The genome visualization tool CIRCOS was used to display the chromosomal location of *GhZFP* genes. The chromosome number is shown on the top of each chromosome. The scale bar indicates the length in megabases (Mb)

To determine the chromosomal location of *GhZFP* genes in *G. hirsutum*, the physical distribution of *GhZFP* genes along the chromosomes was performed using positional information files downloaded from the CottonGen website. Among the 29 *GhZFP* genes, 22 genes deposited on 17 chromosomes, including nine chromosomes from the At subgenome and eight from the Dt subgenome (Fig. 2). Most of the chromosomes possessed only one *GhZFP* gene, except for the At_04 and At_09 chromosomes. Among the *GaZFP* and *GrZFP* genes, 23 members from *G. arboreum* were distributed on 10 chromosomes and 17 members from *G. rainomdii* were distributed on nine chromosomes (Additional file 5: Figure S4).

We further investigated possible *GhZFP* genes created by whole-genome duplications (WGD). Among all of the *GhZFP* genes, 25 were produced from WGD and 4 from dispersed duplication events (Additional file 1: Table S2), indicating that WGD events played an important role in the expansion of *GhZFP* gene. Additionally, in order to investigate the evolutionary history of *GhZFP* genes, the KaKs_calculator 2.0 program was used to calculate synonymous and non-synonymous substitution rates. The Ks ratios of *GhZFP* gene pairs ranged from 0.016 to 0.555. The approximate time of duplication events of *GhZFPs* ranged from 0.53 million years ago (MYA) to 18.5 MYA (Table 2).

**Table 2:** Dates of duplication for the duplicated gene pairs

Gene 1	Gene 2	Length	Ks	T = Ks/2λ
GhZFP3–4	GhZFP33	876	0.026	0.87
GhZFP3–2	GhZFP3–1	759	0.016	0.53
GhZFP10–8	GhZFP10–3	684	0.041	1.37
GhZFP10–11	GhZFP1012	804	0.022	0.73
GhZFP10–7	GhZFP10–6	645	0.019	0.63
GhZFP10–2	GhZFP10–1	885	0.024	0.8
GhZFP8–2	GhZFP84	717	0.555	18.5
GhZFP5–1	GhZFP4–1	717	0.046	1.53

### Gene structure and conserved motifs of GhZFP proteins

**Fig. 3:**
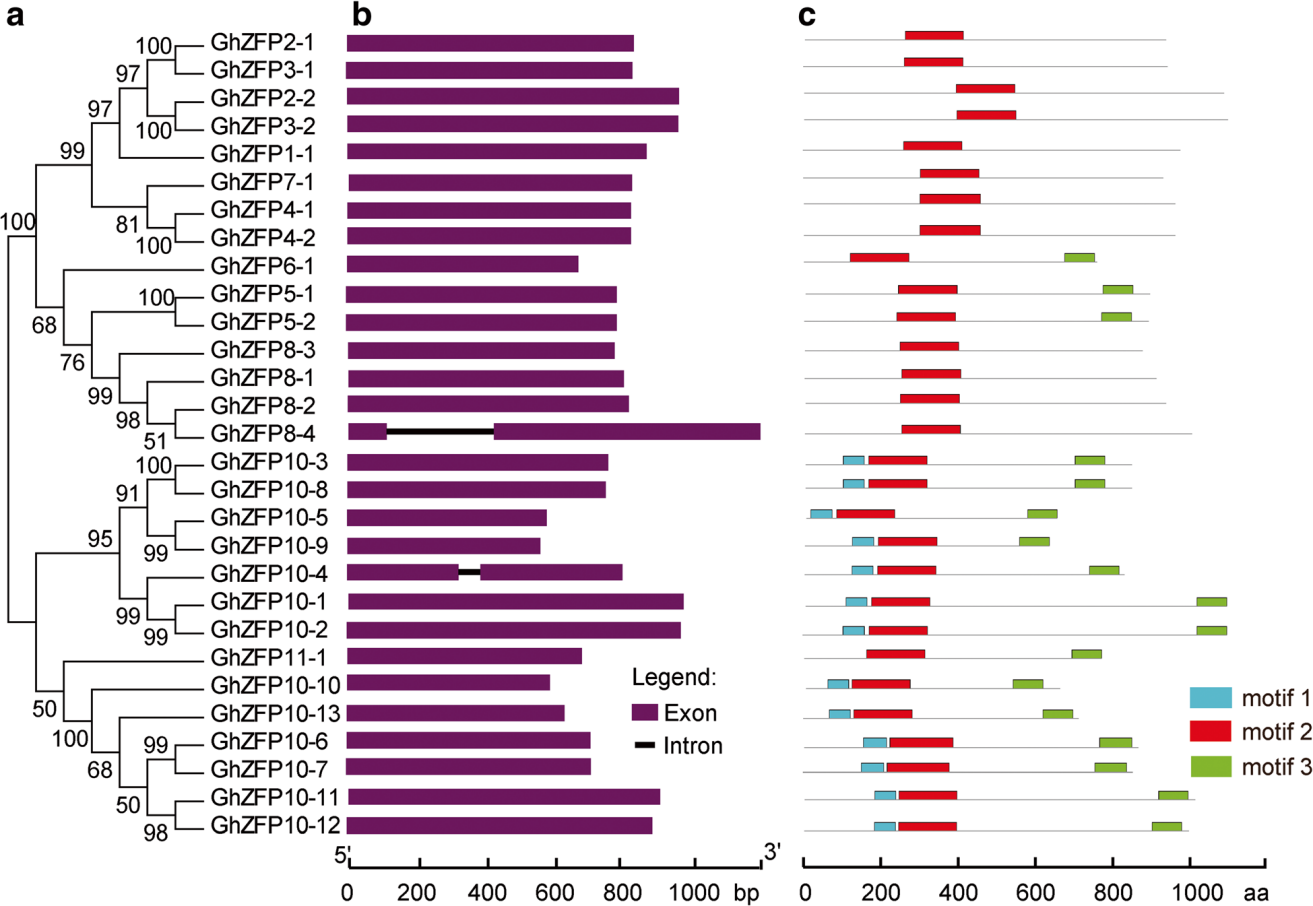
Phylogenetic relationships, gene structures and protein domain architecture of *GhZFP* genes**. a** Phylogenetic relationships between GhZFPs. A phylogenetic tree was generated using the maximum likelihood method with 1,000 bootstrap iterations in MEGA 6.0 software. **b** Gene structure (exon-intron organization) analysis of *GhZFPs*. Exons and introns are represented by purple boxes and black lines, respectively. The scale bar is shown at the bottom. **c** The protein domain architecture of *GhZFP* genes. The number and order of motifs in each *GhZFP* genes are shown. Motif 2 in (c) is the ZFP domain

With the aim of gaining better understanding of the similarity and diversity between different members of GhZFP proteins, we generated an unrooted phylogenetic tree with the deduced GhZFP amino acid sequences (Fig. 3a) and performed a comparative analysis of exon-intron structure. We found that the gene length of *GhZFP* was relatively conserved, with *GhZFP8–4* having the longest (1.2 Kb) and *GhZFP10–9* having the shortest (0.6 Kb) genomic sequence (Fig. 3b). Twenty seven out of 29 *GhZFP* genes had no introns, except for *GhZFP8–4* and *GhZFP10–4,* which contained one intron. These results were quite similar to the structure of *ZFP* genes in *Populus trichocarpa* [35]. Most *GhZFPs* within the same subclades exhibited similar gene structure in terms of numbers and lengths of introns and exons, which was consistent with subfamilies in the phylogenetic tree (Fig. 3a).

Furthermore, we investigated the conserved motifs in GhZFP proteins to understand the diversity of motif compositions among GhZFP proteins. A total of three conserved motifs, named motif 1 to motif 3, were identified in GhZFP proteins. The number of conserved motifs in each GhZFP varied from 2 to 3 and most GhZFPs within the same subfamily exhibited similar motif compositions (Fig. 3c). In addition, most GhZFP proteins possessed three conserved motifs, except for GhZFP1, GhZFP2, GhZFP3, GhZFP4 and GhZFP8 subfamilies, which contained two conserved motifs, indicating that GhZFP proteins showed functional divergence.

### *GhZFP8* subfamily genes were highly expressed during fiber cell development

To determine which *GhZFP* genes potentially function in fiber cell development, the expression profiles of individual genes were investigated using transcriptome data from different developmental stages of fiber cells, including fibers at 0, 3, 10, and 15 DPA.

**Fig. 4:**
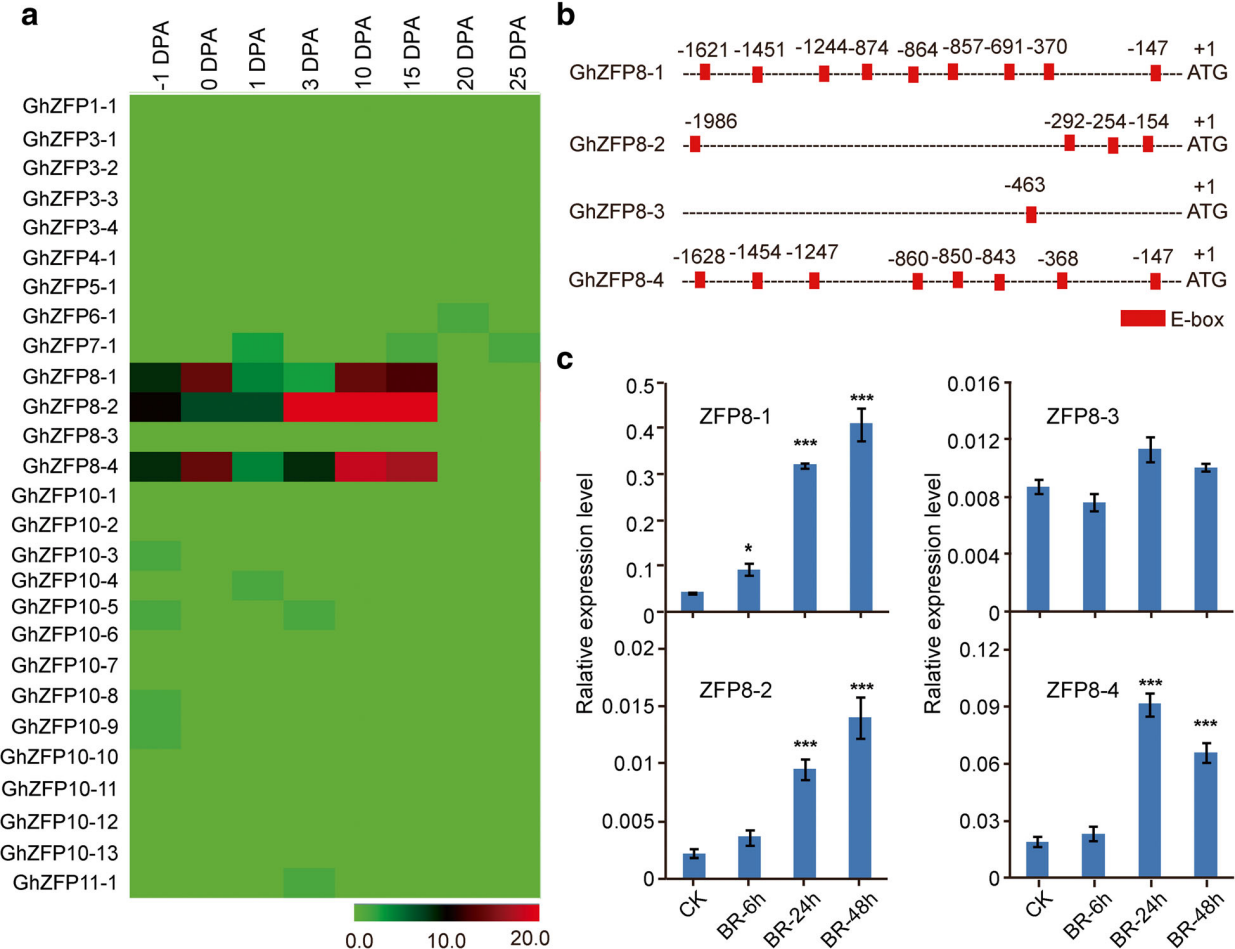
Expression profiles of *GhZFP* genes during fiber development**. a** RNA-seq data heat map of *GhZFP* gene expression levels during different stages of fiber growth. The differences in gene expression are shown in different colors. **b** *Cis*-element analysis of *GhZFP8* promoters. E-box (CANNTG) motifs (red) were predicted using the online toolkit, PLACE. A total of 2,000 bp promoter sequence for each *GhZFP8* were used for the analysis. **c** Exogenous BR promotes the transcription of *GhZFP8*. Statistical significance was determined using one-way analysis of variance combined with Tukey’s test. *P < 0.05; **P < 0.01; ***P < 0.001

We found that most of *GhZFP* genes from the same subfamily shared similar expression patterns. Notably, all members of the *GhZFP8* subfamily, except for *GhZFP8–3*, were highly expressed during fiber cell initiation and elongation development (Fig. 4a)*,* suggesting that these genes may be involved in cotton fiber cell development. We extracted 2000 bp sequences upstream of transcription start codon (ATG) as promoter region. When analyzing the promoter regions of *GhZFP8* genes, a large number of E-box elements (CANNTG, where N can be any nucleotide) were found in *GhZFP8* promoter regions (Fig. 4b). E-box elements can be recognized by BES1, a core transcription factor in the BR signaling pathway [36].

Three *GhZFP8* genes, *GhZFP8*–1, *GhZFP8*–2 and *GhZFP8*–4, contained at least four E-box elements in their promoter regions, while *GhZFP8–3* contained only one E-box (Fig. 4b). These results indicate that the expression of *GhZFP8* genes may be induced by BR. To further confirm this, we performed qRT-PCR experiments to assess the transcription levels of *GhZFP8* genes with or without BR treatment in vitro (Fig. 4c). Our results showed that the transcripts of *GhZFP8*–1, *GhZFP8*–2 and *GhZFP8*–4 were significantly induced after BR application for 24 h (Fig. 4c). However, BR did not induce *GhZFP8*–3 transcripts even when the ovules were treated with BR for 48 h. These results imply that *GhZFP8* may be required for cotton fiber cell development.

### Gene expression analysis of *GhZFP* genes in response to plant hormone treatment

**Fig. 5:**
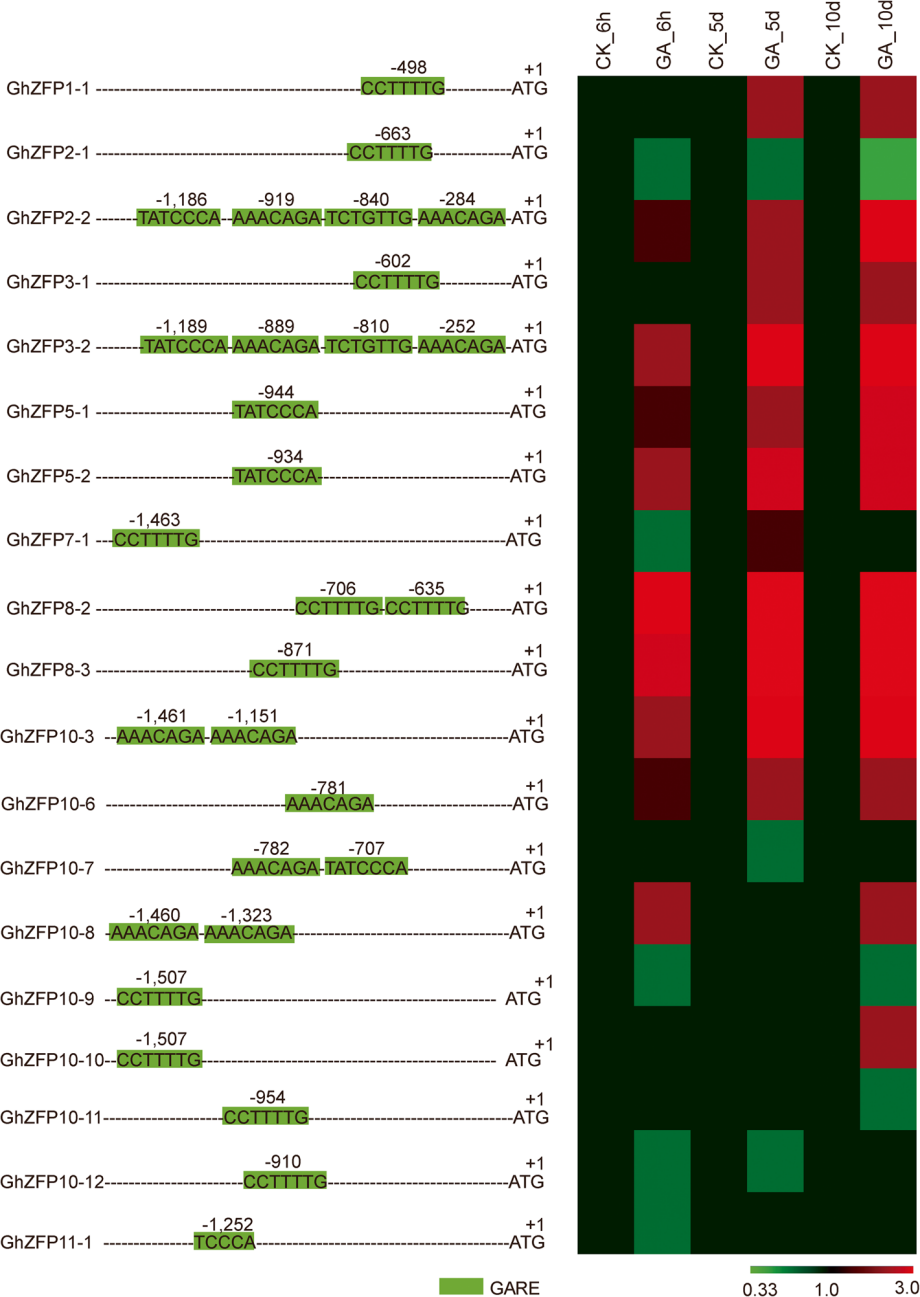
Expression profiles of 19 *GhZFP* genes under GA_3_ treatment**.** Analysis of 19 *GhZFP* genes with GARE elements present in their promoter regions (left). GAREs (green) were predicted using the PLACE website (http://www.dna.affrc.go.jp/htdocs/PLACE/). Fold change of 19 *GhZFP* genes under GA_3_ treatment (right). All treatments were performed with three biological and three technical replicates. The relative gene expression levels were determined using cotton *GhUBQ7* as a control

Phytohormones play pivotal roles in plant development. Considerable evidence suggests that gibberellic acid, auxin and BR are required for cotton fiber cell development [34, 37–39]. In order to explore the relationship between *GhZFP* genes and gibberellin, we analyzed the *cis*-elements in *GhZFP* promoter regions. A large number of GARE elements (CCTTTG or TATCCCA or AAACAGA or TCTGTTG) were found within *GhZFP* promoter regions (Fig. 5). Specifically, 19 out of 29 *GhZFP* genes possessed at least one GARE element. These results strongly suggest that the expression of *GhZFP* genes may be regulated by gibberellic acid. To confirm this finding, expression analysis of *GhZFP* genes was carried out after treatment with gibberellin. Our results showed that a total of 15 *GhZFP* genes were responsive to gibberellin treatment (Fig. 5), except for *GhZFP2–1*, *GhZFP7–1*, *GhZFP10–7* and *GhZFP11–1* genes.

**Fig. 6:**
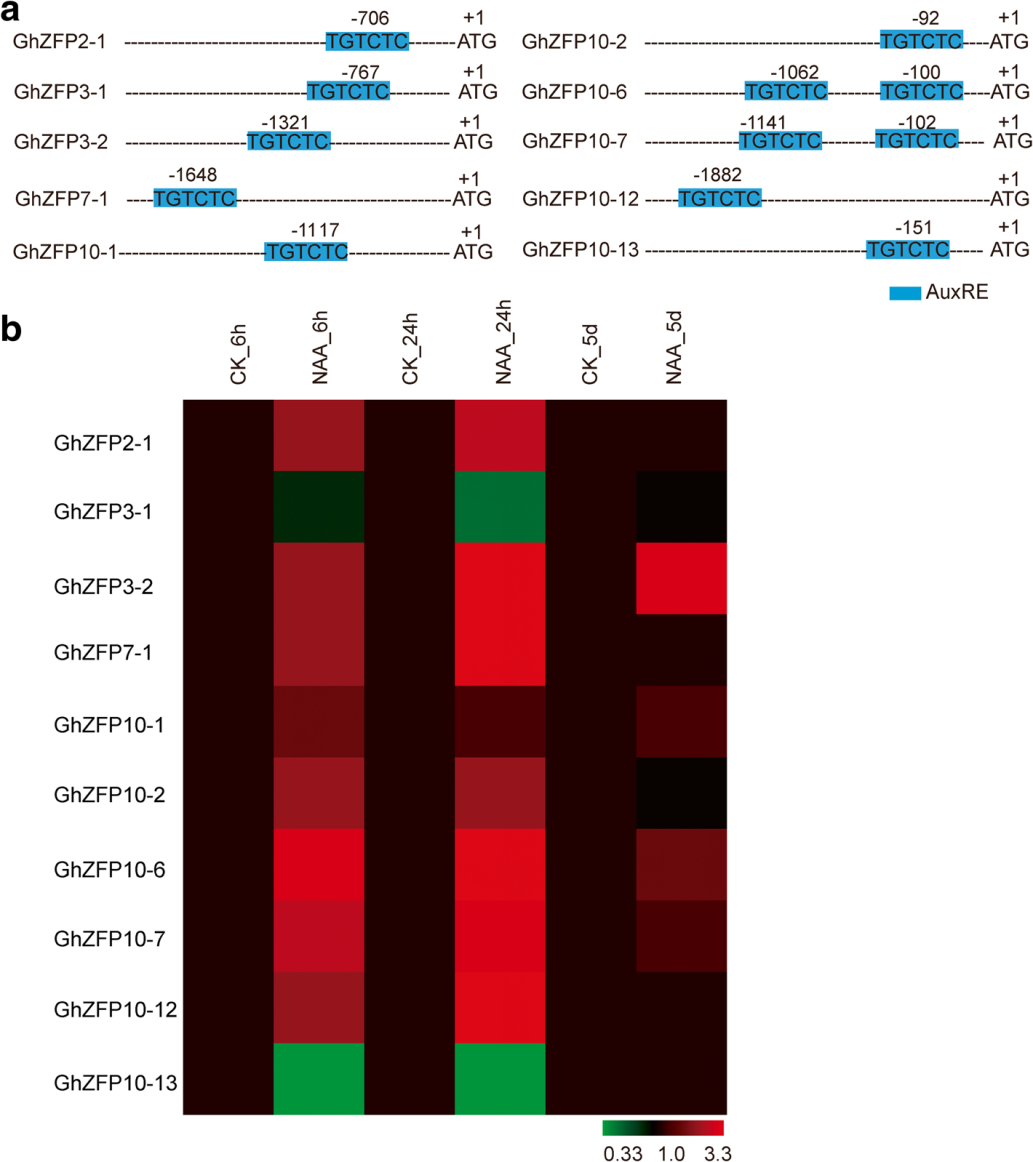
Expression profiles of 10 *GhZFP* genes under NAA treatment. **a** Analysis of 10 *GhZFP* with AuxRE elements in their promoter regions. AuxREs (blue) were predicted using the PLACE website. **b** NAA activated transcription of most *GhZFP* genes with AuxRE elements in their promoter regions. Relative expression levels of each gene were determined after normalizing to the expression level in CK (no chemical added) ovules, which was set to 1.0

Auxin is also known to play important roles in promoting cotton fiber cell development. Overexpression of the auxin biosynthetic gene *iaaM* significantly increased fiber cell initiation [38]. We then identified the *GhZFP* genes responsive to auxin. To this end, we used a similar method to identify genes that were responsive to gibberellic acid. Auxin response factors (ARF), key components in the auxin signaling pathway, specifically recognize and bind to auxin responsive elements (AuxRE, TGTCTC) to regulate downstream auxin responsive genes [40]. Our data showed that a total of 10 *GhZFP* genes were found to contain AuxRE elements in their promoter regions (Fig. 6a). qRT-PCR analysis showed that the transcript levels of seven *GhZFP* genes significantly increased after application of 5 μM NAA, an auxin analog, for 24 h (Fig. 6b). These results suggest that auxin may promote the expression of *GhZFP* genes.

**Fig. 7:**
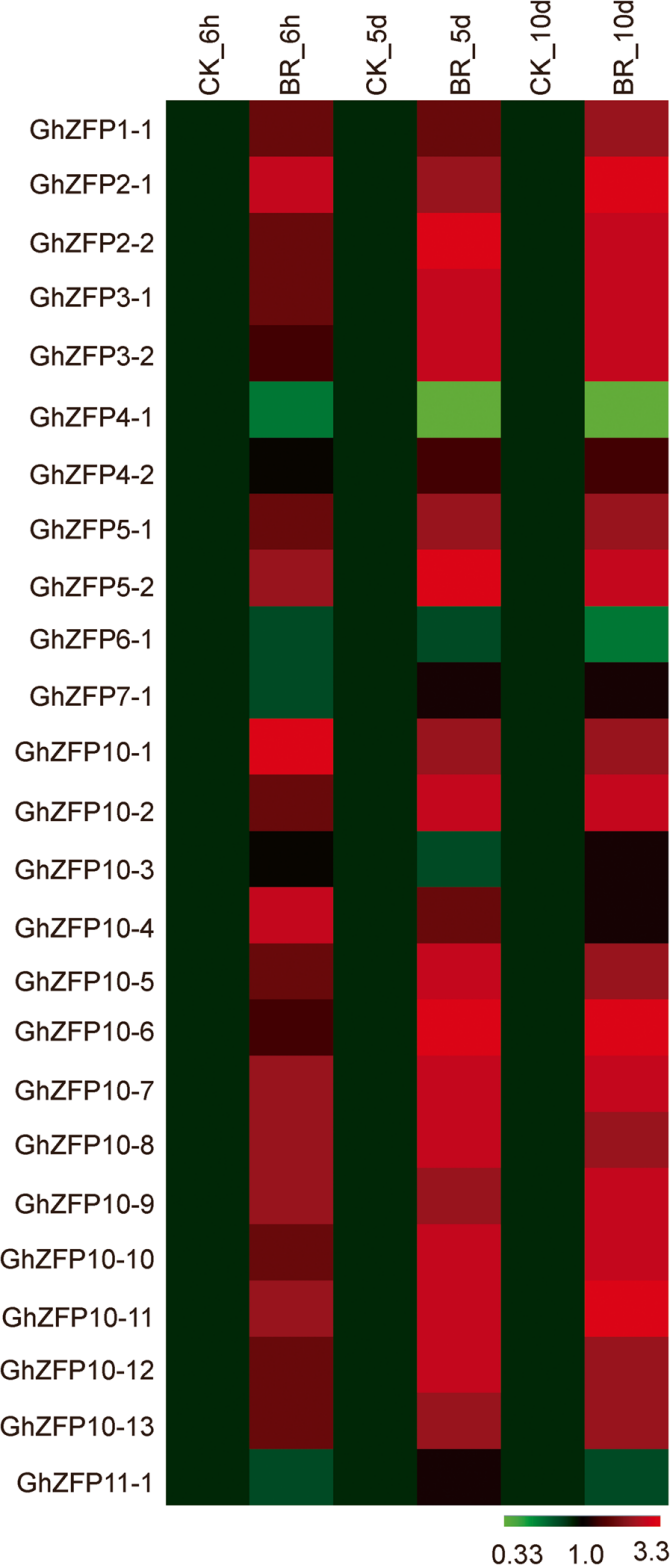
Heat map of expression levels of *GhZFP* genes with the BES1 element in their promoter regions under BR treatment. Differences in gene expression are shown in different colors indicated in the scale. All treatments were performed with three biological and three technical replicates

BR is also involved in fiber growth and exogenous application of BR in vitro promoted fiber cell elongation [33, 37]. E-box elements are specifically recognized by the BES1 transcription factor, which plays a key role in BR-regulated gene expression [36]. We analyzed the distribution of E-box elements in *GhZFP* promoter regions and assayed the transcription levels of the *GhZFP* genes with or without BR treatment. Surprisingly, 25 out of 29 *GhZFP* genes contained an E-box element in their promoter regions (Additional file 6: Figure S5), indicating that *GhZFP* transcripts may be induced by BR. Further evidence was found in significantly stimulated transcription levels for the majority of *GhZFP* genes with an E-box element in their promoter regions when they were treated with 5 μM BR (Fig. 7).

Taken together, transcription of *GhZFP* genes was widely induced by gibberellic acid, auxin and BR, suggesting that these genes play important roles in phytohormone regulation of cotton development.

## Discussion

The ZFP gene family is one of the largest gene families and its members are involved in a wide range of functions in plant growth and development [41, 42]. In *Arabidopsis*, there are 211 zinc finger proteins, which constitute the most abundant family of putative transcriptional regulators in plants [42]. Among them, only 10 members contain just a single zinc finger domain, for which they are named ZFP proteins. *ZFP* family genes have been studied in *Arabidopsis*, rice and petunia [42–44]. However, the *ZFP* gene family has not been investigated in *G. hirsutum*. In this study, we identified 29 *GhZFP* genes in *G. hirsutum,* including 13 genes from the At subgenome and 16 from the Dt subgenome (Additional file 1: Table S1). Notably, 23 *ZFP* genes were found in *G. arboreum* and *G. raimondii*, each, suggesting that gene loss events have occurred in the *ZFP* gene family after the polyploidization in *G. hirsutum*, which also confirmed prior work that a large number of gene loss events occurred in allotetraploid cotton [45, 46].

Gene duplication events are involved in gene expansion and genomic realignments [47]. Gene duplication contributed to functional innovation and expansion of genes, especially for transcription factor gene families in plants [35]. To investigate the origin of *ZFP* genes in *G. hirsutum*, we analyzed their distribution along the chromosomes and gene duplication events. Among the 29 *GhZFP* genes, 22 members were located across 17 chromosomes (Fig. 2). Exon-intron organization, or the genomic structure of genes, plays an important role in the evolution of gene families and gene splicing [48]. Our results showed that all of the *GhZFP* genes had only one exon, except for *GhZFP8–4* and *GhZFP10–4*, which had one intron between two exons, respectively (Fig. 3). This may be due to incomplete cDNA molecules recombining with their genomic copies and horizontal transfer, which are found to lead to the loss of introns within genomes [49–51]. In *Populus trichocarpa*, most of the C2H2-ZF group I genes had no introns [35], consistent with the exon-intron arrangement in *G. hirsutum*.

To better understand the role of *GhZFP* genes in *G. hirsutum*, we performed a systematic analysis on gene expression patterns throughout several developmental stages of cotton fiber cells. The transcriptome data were used to analyze the expression levels of *GhZFP* genes. Interestingly, most *GhZFP8* subfamily genes were significantly expressed in fiber cells (Fig. 4a), strongly suggesting the involvement of *GhZFP8* genes in fiber development. We also performed *cis*-element prediction on *GhZFP8* promoter regions to explore the mechanisms of *GhZFP8* regulating fiber growth. We found abundant E-box elements in *GhZFP8* promoter regions. Since BES1 is reportedly part of the BR response pathway, this finding suggests that transcription of *GhZFP8* genes could be activated by BR. The phytohormone, BR, plays an important role in regulating fiber development [33, 52]. Our findings suggest that BR could induce *GhZFP8* expression to regulate fiber development.

Auxin, gibberellic acids and BR play important roles in fiber development [33, 37–39]. In order to explore the link between *GhZFP* genes and these hormones in detail, we investigated the transcriptional expression levels of *GhZFP* genes before and after treatment with the individual hormones. When analyzing the promoter regions of all *GhZFP* genes, 19 of them were found to possess GARE elements. Exogenous application of GA_3_ significantly induced the transcription of most *GhZFP* genes with GARE elements in their promoter regions (Fig. 5). Applications of NAA, an auxin analog, resulted in significant transcriptional up-regulation of seven genes which contained AuxRE elements in their promoters (Fig. 6). Among the 29 *GhZFP* genes, 25 members contained an E-box element in their promoter region, and most of which were up-regulated after treatment with BR for 5 days (Fig. 7). Our findings suggest that *GhZFP* genes were widely induced by auxin, gibberellin and BR, which provides a foundation for the identification of more downstream genes with potential roles in phytohormone stimuli, and a basis for breeding better cotton varieties in the future.

## Conclusions

Our study provides a comprehensive analysis of the *GhZFP* gene family. We revealed that *GhZFP8* genes were involved in cotton fiber development. This study will expand our understand the precise role of *GhZFP* genes in cotton fiber development and in adaption to phytohormone stimuli. Our findings will also further provide clues for breeding better cotton varieties in the future.

## Additional files

Additional file 1: **Table S1.** Analysis of *G. hirsutum ZFP* gene family and its orthologs in AA and DD cotton genomes. **Table S2.** Analysis of duplication events in *G. hirsutum* ZFP genes located in chromosomes. **Table S3.** A list of primers used in this study. (PDF 112 kb)

Additional file 2: **Figure S1.** Phylogenetic analysis of the *ZFP* gene family**.** MEGA 6.0 software was used with the maximum likelihood method and bootstrapping with 1,000 iterations. At, *Arabidopsis thaliana*; Ga, *Gossypium arboreum*; Gr, *Gossypium raimondii*; Gh, *Gossypium hirsutum*. (PDF 165 kb)

Additional file 3: **Figure S2.** Phylogenetic analysis of the *ZFP* gene family. MEGA 6.0 software was used with the Neighbor-Joining method and bootstrapping with 1,000 iterations. (PDF 253 kb)

Additional file 4: **Figure S3.** Phylogenetic analysis of the *ZFP* gene family. The phylogenetic tree was constructed using the full length ZFP protein amino acid sequences. At, *Arabidopsis thaliana*; Ga, *Gossypium arboreum*; Gr, *Gossypium raimondii*; Gh, *Gossypium hirsutum*; Tc*, Theobroma cacao*; BGIOSGA, *Oryza sativa*; Glyma, *Glycine max*. (PDF 515 kb)

Additional file 5: **Figure S4.** Chromosomal location of *GaZFP* (a) and *GrZFP* (b) genes on chromosomes. The chromosome number is shown on the top of each chromosome. The scale bar indicates the length in megabases (Mb). (PDF 199 kb)

Additional file 6: **Figure S5.** Analysis of 25 *GhZFP* with BES1 element present in their promoter regions. BES1 elements (red) were predicted using the online PLACE website. (PDF 424 kb)

## Data Availability

The cotton genome data analyzed during this article is available from CottonGen (https://www.cottongen.org) and the *A. thaliana* genome sequence is accessible at TAIR 10 (http://www.arabidopsis.org).

## Abbreviations

ARF: Auxin responsive factors
BR: Brassinosteroids
DPA: Day post anthesis
GFF: General Feature Format
GSDS: Gene Structure Display Server
MARD1: The mediator of ABA-regulated dormancy1
MEME: Multiple Expectation maximization for Motif Elicitation
MYA: Million years ago
RD21A: Responsive to dehydration 21A
WGD: Whole genome duplication
