# Supplementary material for: Comprehensive analyses of ZFP gene family and characterization of expression profiles during plant hormone response in cotton
Source: BMC Plant Biol. 2019 Jul 23;19:329. doi: 10.1186/s12870-019-1932-6 (PMC6652020; doi:10.1186/s12870-019-1932-6)
Supplement: Supplementary file 1 — Table S1. Analysis of G. hirsutum ZFP gene family and its orthologs in AA and DD cotton genomes. Table S2. Analysis of duplication events in G. hirsutum ZFP genes located in chromosomes. Table S3. A list of primers used in this study. (PDF 112 kb) [file 12870_2019_1932_MOESM1_ESM.pdf]

Table S1. Analysis of *G. hirsutum ZFP* gene family and its orthologs in AA and DD cotton genomes.

GeneID	Length of amino acid	Gene name	Orthologous	Length of amino acid	Genename
CotAD_24466	263	GhZFP1-1	Cotton_A_35683	261	GaZFP1-1
CotAD_10965	253	GhZFP2-1	NA	NA	NA
CotAD_00109	293	GhZFP2-2	Cotton_A_01186	270	GaZFP2-2
CotAD_06894	253	GhZFP3-1	Cotton_A_09094	253	GaZFP3-1
CotAD_40989	253	GhZFP3-2	NA	NA	NA
CotAD_58159	250	GhZFP4-2	Cotton_A_30668	250	GaZFP4-2
CotAD_52379	239	GhZFP5-1	NA	NA	NA
CotAD_55461	239	GhZFP5-2	Cotton_A_19328	239	GaZFP5-2
CotAD_24947	204	GhZFP6-1	Cotton_A_05952	204	GaZFP6-1
NA	NA	NA	Cotton_A_30896	241	GaZFP6-2
CotAD_24229	250	GhZFP7-1	Cotton_A_28213	202	GaZFP7-1
CotAD_75509	243	GhZFP8-1	Cotton_A_40927	243	GaZFP8-1
NA	NA	NA	Cotton_A_40319	248	GaZFP8-2
NA	NA	NA	Cotton_A_38190	295	GaZFP10-1
NA	NA	NA	Cotton_A_05560	246	GaZFP10-2
NA	NA	NA	Cotton_A_00942	228	GaZFP10-3
NA	NA	NA	Cotton_A_25087	171	GaZFP10-7
NA	NA	NA	Cotton_A_07903	198	GaZFP10-8
NA	NA	NA	Cotton_A_25086	206	GaZFP10-9
CotAD_32787	178	GhZFP10-10	Cotton_A_23121	178	GaZFP10-10
CotAD_41655	270	GhZFP10-12	Cotton_A_07313	270	GaZFP10-12
NA	NA	NA	Cotton_A_24729	191	GaZFP11-1
NA	NA	NA	Cotton_A_28643	200	GaZFP11-2
NA	NA	NA	Cotton_A_31377	165	GaZFP11-3
NA	NA	NA	Cotton_A_00011	206	GaZFP11-5
NA	NA	NA	Cotton_D_gene_10023188	261	GrZFP1-1
NA	NA	NA	Cotton_D_gene_10022380	253	GrZFP2-1
NA	NA	NA	Cotton_D_gene_10015323	271	GrZFP3-1
NA	NA	NA	Cotton_D_gene_10037091	293	GrZFP3-2
CotAD_47390	250	GhZFP4-1	Cotton_D_gene_10030397	116	GrZFP4-1
NA	NA	NA	Cotton_D_gene_10009040	399	GrZFP5-1
NA	NA	NA	Cotton_D_gene_10001715	349	GrZFP6-1
NA	NA	NA	Cotton_D_gene_10024530	318	GrZFP7-1
CotAD_42933	248	GhZFP8-2	Cotton_D_gene_10036444	400	GrZFP8-2
CotAD_43757	235	GhZFP8-3	Cotton_D_gene_10006192	235	GrZFP8-3
CotAD_47769	269	GhZFP8-4	NA	NA	NA
CotAD_50182	295	GhZFP10-1	Cotton_D_gene_10004243	490	GrZFP10-1
CotAD_18982	295	GhZFP10-2	Cotton_D_gene_10026033	456	GrZFP10-2
CotAD_15364	228	GhZFP10-3	NA	NA	NA
CotAD_30572	224	GhZFP10-4	NA	NA	NA
CotAD_31112	176	GhZFP10-5	Cotton_D_gene_10021529	238	GrZFP10-5
CotAD_36452	215	GhZFP10-6	Cotton_D_gene_10037780	215	GrZFP10-6
CotAD_16589	216	GhZFP10-7	NA	NA	NA
CotAD_07166	228	GhZFP10-8	Cotton_D_gene_10024620	228	GrZFP10-8
CotAD_31113	170	GhZFP10-9	Cotton_D_gene_10021528	170	GrZFP10-9
NA	NA	NA	Cotton_D_gene_10009865	221	GrZFP10-10
CotAD_11415	274	GhZFP10-11	Cotton_D_gene_10015481	272	GrZFP10-11
CotAD_17560	192	GhZFP10-13	Cotton_D_gene_10024366	190	GrZFP10-13
NA	NA	NA	Cotton_D_gene_10001027	198	GrZFP11-2
NA	NA	NA	Cotton_D_gene_10005248	171	GrZFP11-4
NA	NA	NA	Cotton_D_gene_10017952	342	GrZFP11-3
CotAD_24782	207	GhZFP11-1	Cotton_D_gene_10021164	192	GrZFP11-1

Table S2. Analysis of duplication events in *G. hirsutum ZFP* genes located in chromosomes.

Gene ID	Gene name	Duplication type
CotAD_24466	GhZFP1-1	Whole Genome/Segmental Duplication
CotAD_10965	GhZFP2-1	Whole Genome/Segmental Duplication
CotAD_00109	GhZFP2-2	Whole Genome/Segmental Duplication
CotAD_06894	GhZFP3-1	Whole Genome/Segmental Duplication
CotAD_40989	GhZFP3-2	Whole Genome/Segmental Duplication
CotAD_47390	GhZFP4-1	Whole Genome/Segmental Duplication
CotAD_58159	GhZFP4-2	Whole Genome/Segmental Duplication
CotAD_52379	GhZFP5-1	Whole Genome/Segmental Duplication
CotAD_55461	GhZFP5-2	Whole Genome/Segmental Duplication
CotAD_24229	GhZFP7-1	Whole Genome/Segmental Duplication
CotAD_75509	GhZFP8-1	Whole Genome/Segmental Duplication
CotAD_42933	GhZFP8-2	Whole Genome/Segmental Duplication
CotAD_43757	GhZFP8-3	Whole Genome/Segmental Duplication
CotAD_47769	GhZFP8-4	Whole Genome/Segmental Duplication
CotAD_50182	GhZFP10-1	Whole Genome/Segmental Duplication
CotAD_18982	GhZFP10-2	Whole Genome/Segmental Duplication
CotAD_30572	GhZFP10-4	Whole Genome/Segmental Duplication
CotAD_31112	GhZFP10-5	Whole Genome/Segmental Duplication
CotAD_36452	GhZFP10-6	Whole Genome/Segmental Duplication
CotAD_16589	GhZFP10-7	Whole Genome/Segmental Duplication
CotAD_07166	GhZFP10-8	Whole Genome/Segmental Duplication
CotAD_11415	GhZFP10-11	Whole Genome/Segmental Duplication
CotAD_41655	GhZFP10-12	Whole Genome/Segmental Duplication
CotAD_17560	GhZFP10-13	Whole Genome/Segmental Duplication
CotAD_24782	GhZFP11-1	Whole Genome/Segmental Duplication
CotAD_24947	GhZFP6-1	dispersed
CotAD_15364	GhZFP10-3	dispersed
CotAD_31113	GhZFP10-9	dispersed
CotAD_32787	GhZFP10-10	dispersed

Table S3. A list of primers used in this study.

Gene name	Forward primer	Reverse primer
GhZFP1-1	GGCATAATGAATGGTCTAGA	GTGTTGAAAGGGTTAACACT
GhZFP2-1	ACGTCATAGTTGGTCAAGAC	CTTCCTGGTTAACATTCAGA
GhZFP2-2	CCTCCTATAGACCAACAACC	GCATCTCTTCCTTCTTAGGC
GhZFP3-1	CGTCATAGTTGGTCAAGACC	CCTGGTTAACATTCAGACTG
GhZFP4-1	GAACACCAGACACCAGAAAC	CCAAGTCCACTGGTTCTTTA
GhZFP5-1	GCTGATTTCACTCCTTACGA	AGTGGAAGAAGAAGGCTTGA
GhZFP6-1	CATCGTATATGTTGCAAGGT	ACGATCCTAGACGAAGATGT
GhZFP7-1	AGATACAAGAAGTGGGGCAC	GGTGTTGCCATTGCTATAAA
GhZFP8-1	TACGGTTCAAAGAAACTCAT	GTAGATCCAAACTCACATGG
GhZFP8-2	TGGGGTTATGGAGAATTCCT	TTGGCTTGGATTCATAAACG
GhZFP8-3	TGGAACAATACCATTAACGC	ACTCTCGGAACTTGAATCAC
GhZFP8-4	CTATCCCGACCTTTCATCAT	TCACTCGTGAGAAAGTTTCA
GhZFP10-1	AGCAGATTATGTCCAAACCG	TAGGGCTAAGCTCAGACACC
GhZFP10-3	GACAGCATCAAGGGCTATTA	ACTTCACCTTTGGTTGAACA
GhZFP10-4	TTGACAGCTGACAGATATGG	TGTTGTTGTCTTCATCAGCT
GhZFP10-5	GAAGCAGCATGCACTTTAGA	AAGTTCAAGAACCTGCCATT
GhZFP10-6	TCTCCATCTATGAACCCATG	GTCGAAGCTCTAGATCAAGC
GhZFP10-9	AAAGGACAGTCATTTGGGTC	CCTAAGCTCAAGGTCCAAGT
GhZFP10-10	GCCCTAATGGTCTTTTCTTC	TCTATGCCCTAATCGAAGTT
GhZFP10-11	CATCGATTCATCTTCTACCC	CGTCCTTGAATGTACTGCTT
GhZFP10-13	GCTACTGCTATTTCCTCGTC	GCTCCCACCATTAAGATTAC
GhZFP11-1	TCTTACTCTTTCCACCCTCC	CATTAACCAAACCAATCTCC
GhUBQ7	GCATTCCACCTGACCAACA	CGCATTAGGGCACTCTTTT
