# Supplementary material for: Comprehensive analyses of ZFP gene family and characterization of expression profiles during plant hormone response in cotton
Source: BMC Plant Biol. 2019 Jul 23;19:329. doi: 10.1186/s12870-019-1932-6 (PMC6652020; doi:10.1186/s12870-019-1932-6)

Figure S1. Phylogenetic analysis of the *ZFP* gene family in three *Gossypium* species and *A. thaliana* by Maximum Likelihood method.

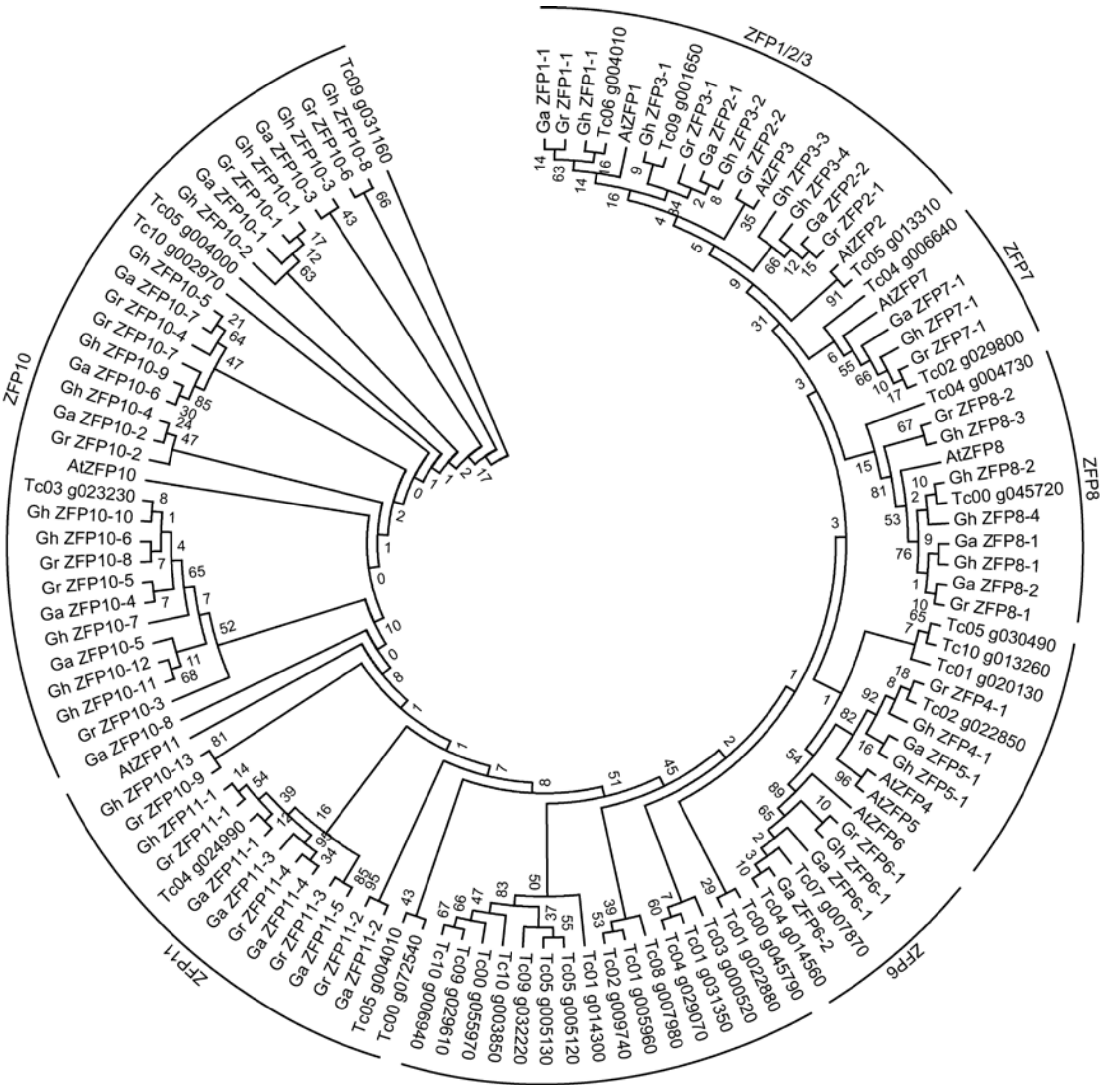

Supplement: Supplementary file 2 — Figure S1. Phylogenetic analysis of the ZFP gene family. MEGA 6.0 software was used with the maximum likelihood method and bootstrapping with 1,000 iterations. At, Arabidopsis thaliana; Ga, Gossypium arboreum; Gr, Gossypium raimondii; Gh, Gossypium hirsutum. (PDF 165 kb) [file 12870_2019_1932_MOESM2_ESM.pdf]
