# Supplementary material for: Comprehensive analyses of ZFP gene family and characterization of expression profiles during plant hormone response in cotton
Source: BMC Plant Biol. 2019 Jul 23;19:329. doi: 10.1186/s12870-019-1932-6 (PMC6652020; doi:10.1186/s12870-019-1932-6)

Figure S2. Phylogenetic analysis of the *ZFP* gene family. MEGA 6.0 software was used with the Neighbor-Joining method and bootstrapping with 1,000 iterations.

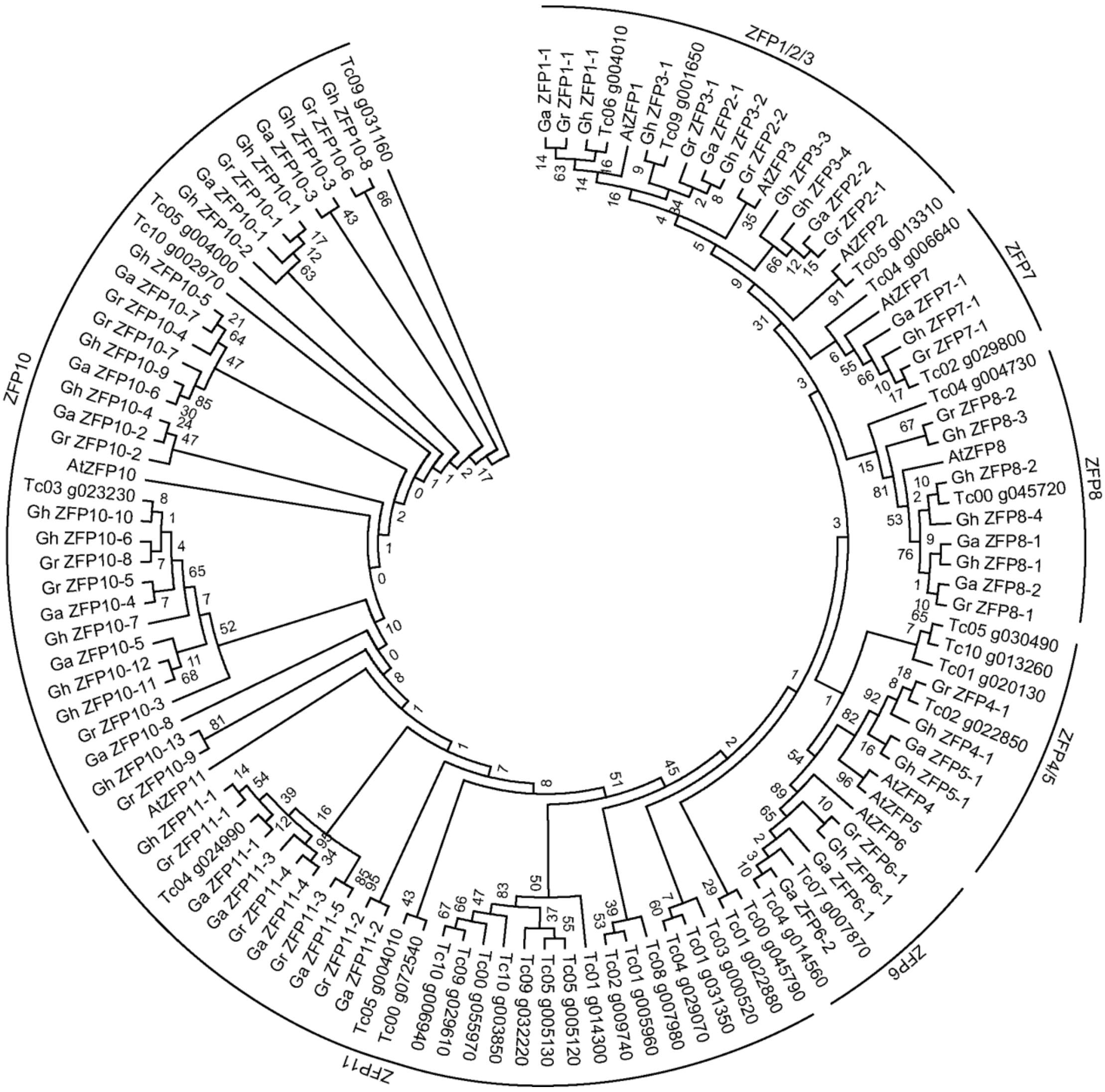

Supplement: Supplementary file 3 — Figure S2. Phylogenetic analysis of the ZFP gene family. MEGA 6.0 software was used with the Neighbor-Joining method and bootstrapping with 1,000 iterations. (PDF 253 kb) [file 12870_2019_1932_MOESM3_ESM.pdf]
