# Supplementary material for: Comprehensive analyses of ZFP gene family and characterization of expression profiles during plant hormone response in cotton
Source: BMC Plant Biol. 2019 Jul 23;19:329. doi: 10.1186/s12870-019-1932-6 (PMC6652020; doi:10.1186/s12870-019-1932-6)

Figure S4. Chromosomal location of *GaZFP* (a) and *GrZFP* (b) genes on chromosomes. The chromosome number is shown on the top of each chromosome. The scale bar indicates the length in megabases (Mb).

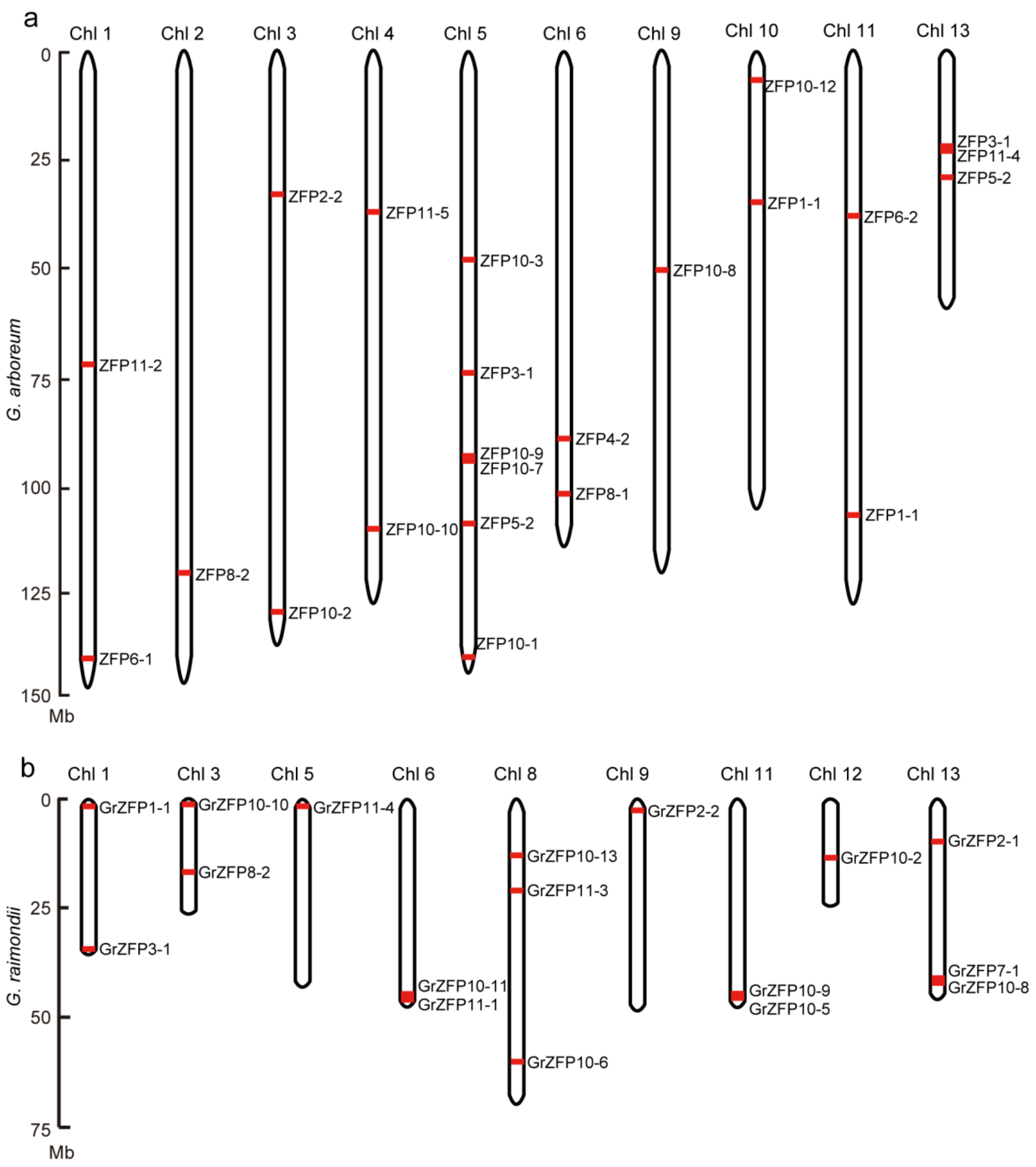

Supplement: Supplementary file 5 — Figure S4. Chromosomal location of GaZFP (a) and GrZFP (b) genes on chromosomes. The chromosome number is shown on the top of each chromosome. The scale bar indicates the length in megabases (Mb). (PDF 199 kb) [file 12870_2019_1932_MOESM5_ESM.pdf]
