# Supplementary material for: Comprehensive analyses of ZFP gene family and characterization of expression profiles during plant hormone response in cotton
Source: BMC Plant Biol. 2019 Jul 23;19:329. doi: 10.1186/s12870-019-1932-6 (PMC6652020; doi:10.1186/s12870-019-1932-6)

Figure S5. Analysis of 25 GhZFP with BES1 element present in their promoter regions. BES1 elements (red) were predicted using the online PLACE website.

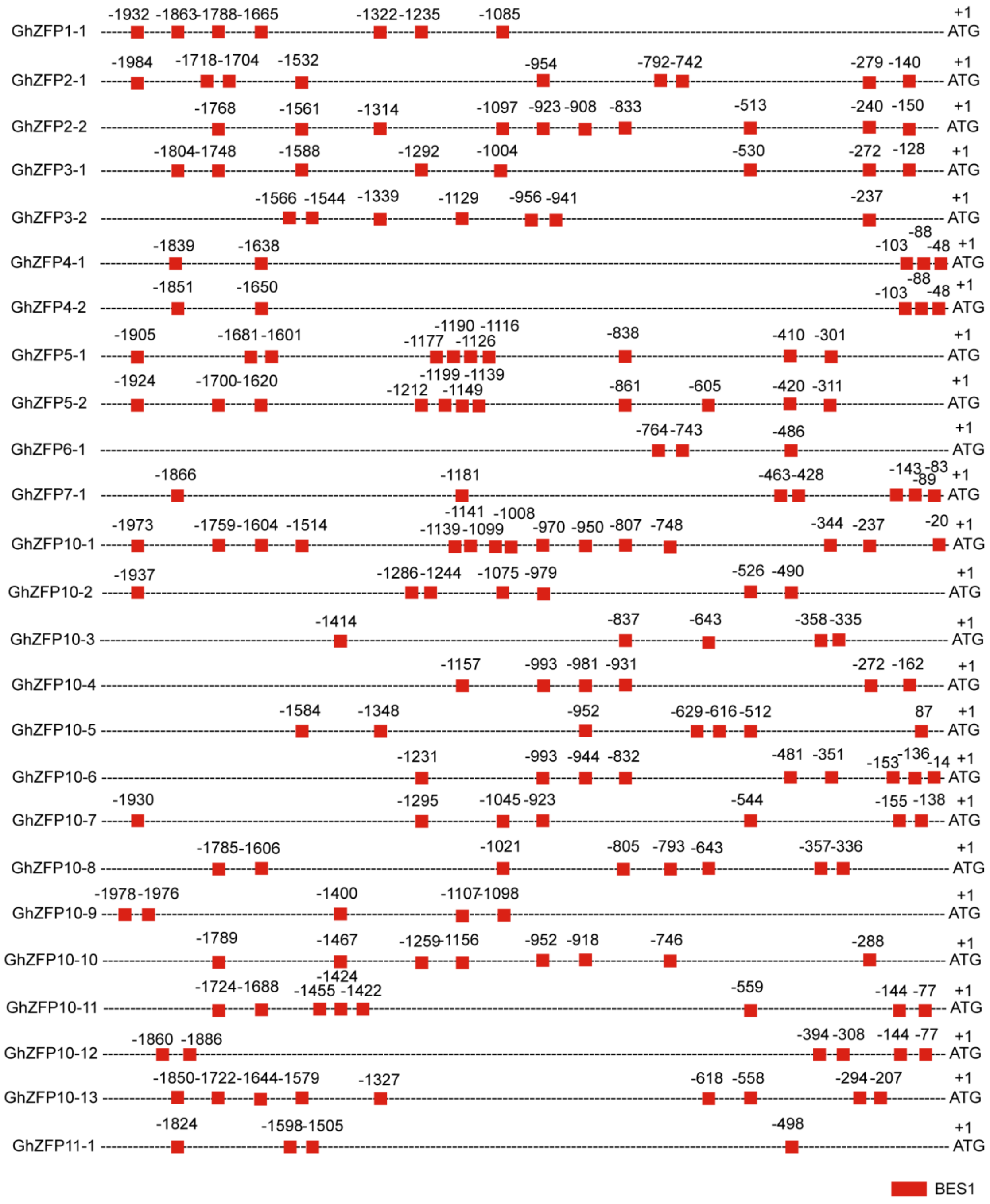

Supplement: Supplementary file 6 — Figure S5. Analysis of 25 GhZFP with BES1 element present in their promoter regions. BES1 elements (red) were predicted using the online PLACE website. (PDF 424 kb) [file 12870_2019_1932_MOESM6_ESM.pdf]
