# Supplementary material for: Comprehensive analyses of ZFP gene family and characterization of expression profiles during plant hormone response in cotton
Source: BMC Plant Biol. 2019 Jul 23;19:329. doi: 10.1186/s12870-019-1932-6 (PMC6652020; doi:10.1186/s12870-019-1932-6)

Figure S3. Phylogenetic analysis of the *ZFP* gene family. The phylogenetic tree was constructed using the full length ZFP protein amino acid sequences. At, *Arabidopsis thaliana*; Ga, *Gossypium arboreum*; Gr, *Gossypium raimondii*; Gh, *Gossypium hirsutum;* Tc*, Theobroma cacao*; BGIOSGA, *Oryza sativa*; Glyma, *Glycine max*.

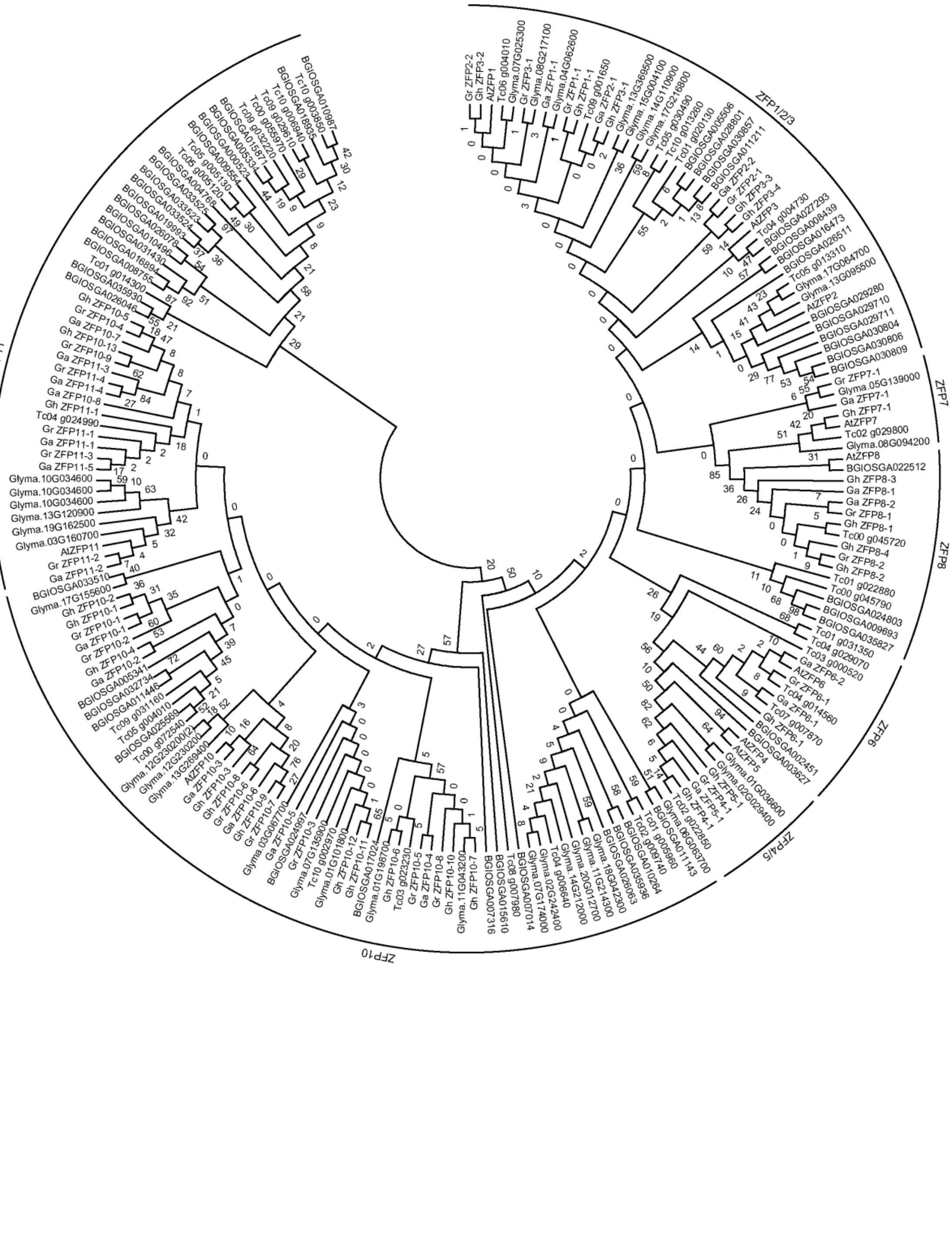

Supplement: Supplementary file 4 — Figure S3. Phylogenetic analysis of the ZFP gene family. The phylogenetic tree was constructed using the full length ZFP protein amino acid sequences. At, Arabidopsis thaliana; Ga, Gossypium arboreum; Gr, Gossypium raimondii; Gh, Gossypium hirsutum; Tc, Theobroma cacao; BGIOSGA, Oryza sativa; Glyma, Glycine max. (PDF 515 kb) [file 12870_2019_1932_MOESM4_ESM.pdf]
